# The Role of TOR1A Polymorphisms in Dystonia: A Systematic Review and Meta-Analysis

**DOI:** 10.1371/journal.pone.0169934

**Published:** 2017-01-12

**Authors:** Vasileios Siokas, Efthimios Dardiotis, Evangelia E. Tsironi, Georgios Tsivgoulis, Dimitrios Rikos, Maria Sokratous, Stylianos Koutsias, Konstantinos Paterakis, Georgia Deretzi, Georgios M. Hadjigeorgiou

**Affiliations:** 1 Department of Neurology, Laboratory of Neurogenetics, University of Thessaly, University Hospital of Larissa, Larissa, Greece; 2 Department of Ophthalmology, Faculty of Medicine, School of Health Sciences, University of Thessaly, Larissa, Greece; 3 Second Department of Neurology, University of Athens, School of Medicine, "Attikon" University Hospital, Athens, Greece; 4 International Clinical Research Center, St. Anne's University Hospital in Brno, Brno, Czech Republic; 5 Department of Vascular Surgery, University Hospital of Larissa, University of Thessalia Medical School, Larissa, Greece; 6 Department of Neurosurgery, University of Thessaly, University Hospital of Larissa, Larissa, Greece; 7 Department of Neurology, Papageorgiou General Hospital, Thessaloniki, Greece; University of Pennsylvania Perelman School of Medicine, UNITED STATES

## Abstract

**Importance:**

A number of genetic loci were found to be associated with dystonia. Quite a few studies have been contacted to examine possible contribution of TOR1A variants to the risk of dystonia, but their results remain conflicting. The aim of the present study was to systematically evaluate the effect of TOR1A gene SNPs on dystonia and its phenotypic subtypes regarding the body distribution.

**Methods:**

We performed a systematic review of Pubmed database to identify all available studies that reported genotype frequencies of TOR1A SNPs in dystonia. In total 16 studies were included in the quantitative analysis. Odds ratios (ORs) were calculated in each study to estimate the influence of TOR1A SNPs genotypes on the risk of dystonia. The fixed-effects model and the random effects model, in case of high heterogeneity, for recessive and dominant mode of inheritance as well as the free generalized odds ratio (OR_G_) model were used to calculate both the pooled point estimate in each study and the overall estimates.

**Results:**

Rs1182 was found to be associated with focal dystonia in recessive mode of inheritance [Odds Ratio, OR (95% confidence interval, C.I.): 1.83 (1.14–2.93), Pz = 0.01]. In addition, rs1801968 was associated with writer’s cramp in both recessive and dominant modes [OR (95%C.I.): 5.99 (2.08–17.21), Pz = 0.00009] and [2.48 (1.36–4.51), Pz = 0.003) respectively and in model free-approach [OR_G_ (95%C.I.): 2.58 (1.45–4.58)].

**Conclusions:**

Our meta-analysis revealed a significant implication of rs1182 and rs1801968 TOR1A variants in the development of focal dystonia and writer’s cramp respectively. TOR1A gene variants seem to be implicated in dystonia phenotype.

## Introduction

Dystonia is a common but heterogeneous movement disorder. It is estimated to be the third most frequent movement disorder worldwide [1]. However, for most dystonia cases the nature and cause remains largely unknown [2]. Increased cortex plasticity through the entire sensorimotor system [3, 4] and functional modifications of the olivo-cerebellar pathway [5] were recognized as endophenotypes of dystonia. Moreover, reduced integrity of cerebello-thalamo-cortical tracts has been observed in symptomatic and asymptomatic carriers of dystonia-linked genes mutations [6].

Recently, a new general definition and a new classification of dystonia have been proposed [7]. Classification is now based on two distinct axes: the etiology and the clinical features, which include age at onset, body distribution, temporal pattern and coexistence of other movement disorders. The widely used term “primary” has been replaced with the term “isolated”, where dystonia is the only motor feature, not counting tremor [7].

TOR1A gene (also known as DYT1) covers an 11k bp region in chr9 and it is consisted of 5 exons. TOR1A protein, called TorsinA belongs to the family of the AAA+ ATPases that can be found in the endoplasmic reticulum and nuclear envelope of most cells [8] including cells of the central nervous system [1] and are associated with a variety of cellular activities [9]. The function of TorsinA and how TOR1A gene mutations lead to dystonia is poorly understood. However, it seems that TorsinA is implicated in several molecular and cellular procedures, such as the interactions between cytoskeleton and membrane, important functions of the endoplasmic reticulum (reaction to stress, secretory pathway, protein degradation, neurites’ expansion) and of the nuclear envelope (membrane formation and cell migration) [1, 8].

Relatively few genetic loci have been identified as potential causing factors of hereditary forms of isolated and combined dystonia, with a wide variation in the mode of inheritance, clinical features, body distribution and age of onset. The DYT1 (TOR1A) form of hereditary dystonia is mainly a generalized (and rarely focal) dystonia, which is inherited by an autosomal dominant mode [10]. Regarding sporadic dystonia, a number of genes have been linked to dystonia phenotypes including but not limited to TOR1A, b-cystathionine synthase (CBS), GTP cyclohydrolase1 (GCH1), dopamine D5 receptor (DRB5) and brain-derive neurotrophic factor (BDNF) genes [11–21]. Moreover, apolipoprotein E (APOE) gene has been reported to modulate the age at onset of primary dystonia [22]. However, TOR1A gene remains the most extensively studied and related to a variety of phenotypes but with conflicting results [23].

Two meta-analyses evaluated, so far, the effects of TOR1A gene variants on primary dystonia [14, 24], whereas there is also one pooled analysis in adult-onset primary focal dystonia [23]. In the first meta-analysis no significant association was found [14], whereas in the second meta-analysis a borderline significant association was reported for the rs1801968 in the subgroup of patients with primary dystonia that also had a positive family history [24]. Similarly, the pooled analysis that examined the effect of rs2296793 and rs1801968 variants on adult-onset primary focal dystonia also did not reveal any significant difference between cases and controls [23].

The previous two meta-analyses [14, 24] used as a clinical outcome endpoint all forms of primary dystonia together without focusing on each dystonia sub-phenotype such as cervical dystonia, blepharospasm or writer’s cramp. Moreover, the pooled analysis of adult-onset primary focal dystonia cases [23] studied only two (rs2296793, rs1801968) of TOR1A gene variants and part of the available studies was used. In the present meta-analysis we aimed to study the effect of all available TOR1A gene SNPs on the risk of dystonia and its sub-phenotypes. Our meta-analysis has included five additional studies that have been published recently and were not included in the previous meta-analyses.

## Methods

### Data extraction

Eligible case-control candidate gene association studies (GAS) were selected by searching Pubmed database. The combination of search strings that was used included the following terms: “dystonia” and “tor1a” and “polymorphism”. The complete search algorithm is available in the **S1 Appendix**. We imposed no language or other restrictions. Last literature search was performed on September 9th, 2016. The reference lists of all retrieved articles were additionally examined to identify studies that may have been missed by the initial database search. The inclusion criteria were a) case-control studies, in which cases were clinically diagnosed of having dystonia and controls were neurologically healthy, b) studies reporting single nucleotide polymorphisms in the TOR1A gene and c) studies that mentioned in the text the genotype frequencies from patients and controls. Studies with incomplete data or without genotype frequencies were excluded from meta-analysis. The initial phenotypic classifications of participants in each study were maintained.

The following information was extracted from each study: author, year of publication, ethnicity of the studied population, numbers of cases and controls, age at disease onset, mean age and gender distribution, genotype frequencies, tested polymorphisms, family history of the participants, method of diagnosis, screening or not of TOR1A ΔGAG mutation, deviation or not from the Hardy-Weinberg Equilibrium (HWE), and tested dystonia phenotypes.

**Fig 1:**
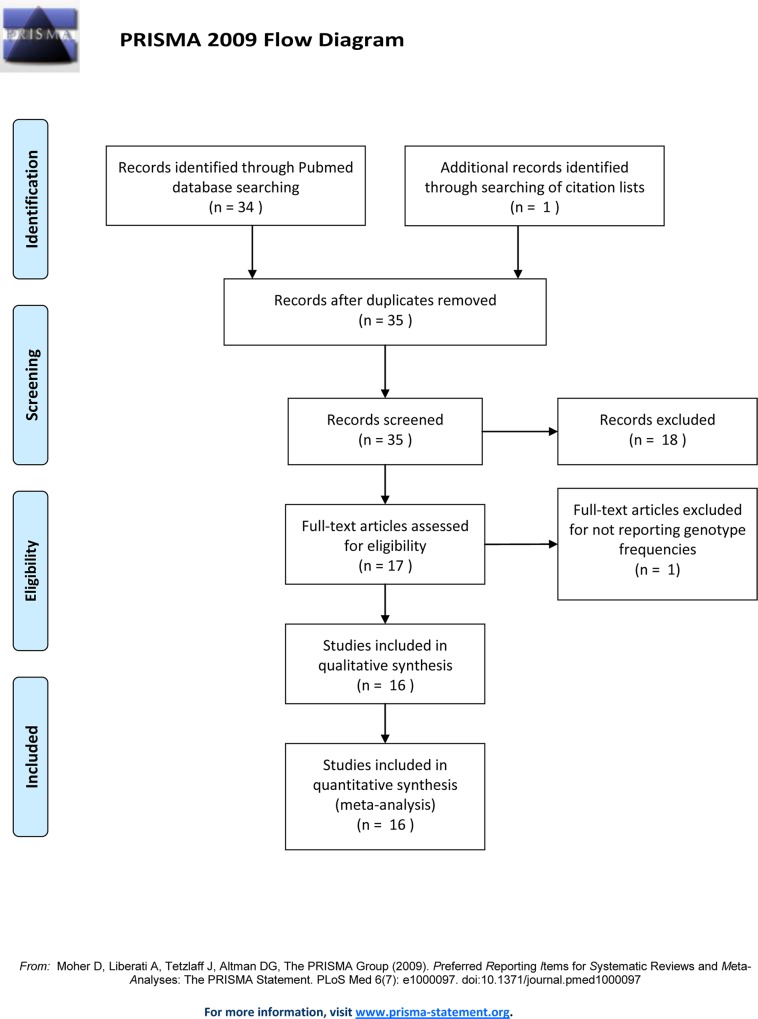
Flow chart presenting the selection of eligible studies.

All individual studies were reviewed and the risk of possible bias was assessed. The flowchart presenting the selection procedure of eligible studies is presented in **Fig 1**.

### Statistical analysis

Dystonia cases were stratified according to the five following phenotypic qualitative traits: a) an overall dystonia group, b) an overall focal dystonia group (containing cervical dystonia, blepharospam, writer’s cramp and other focal dystonias) and the three following focal dystonias’ subgroups c) the cervical dystonia group, d) the blepharospasm group and f) the writer’s cramp group. In each of the previous phenotypic traits, available TOR1A polymorphisms were tested in the meta-analysis. The association between the TOR1A SNPs and the above mentioned dystonia traits was estimated by calculating the pooled odds ratio (OR) and 95% confidence interval (CI) assuming the dominant [(mt/mt+wt/mt) vs (wt/wt)] and the recessive [(mt/mt) vs (wt/mt+wt/wt)] genetic modes of inheritance. The significance of the OR was determined by the Z test (p<0.05 was considered statistically significant). Additionally, the generalized odds ratio (OR_G_) and the corresponding 95%C.I. were applied in order to quantify the association between genotype distribution and disease. OR_G_ is an inheritance model free approach that estimates the mutational load in cases compared to the controls [25, 26]. ORs and OR_G_s with the corresponding 95 C.I. were also calculated in every individual eligible GAS.

The statistical heterogeneity of the studies was calculated with the Cochran’s Q and I^2^ index. The random-effects model (the DerSimonian and Laird method [27]) was applied if there was an indication of substantial heterogeneity (P_Q_ <0.10 and/or I^2^>75%). Otherwise the fixed-effects model (the Mantel-Haenszel method [28]) was used.

Test for possible publication bias was graphically assessed using the funnel plot. Furthermore, it was evaluated by the linear regression asymmetry test by Egger [29], when it was applicable, with p<0.10 considered to be representative of statistically significant publication bias.

All statistical analyses were performed in Review Manager (RevMan) Version 5.2 software [The Nordic Cochrane Centre, The Cochrane Collaboration, Copenhagen, Denmark (http://tech.cochrane.org/revman)]. The OR_G_ was calculated with ORGGASMA (www.biomath.uth.gr) software. The PRISMA guidelines for reporting reviews and meta-analyses (**S2 Appendix**) were applied in this meta-analysis.

## Results

### Study selection and study characteristics

Pubmed database search yielded 34 studies published between September 1997 and March 2016. After title and abstract screening by 2 independent reviewers (VS and ED), 16 potentially eligible studies for the meta-analysis were retained. One additional study was extracted from the references of the identified studies and was included in the meta-analysis [30]. However, one study was excluded from further analysis, as it did not report genotype frequencies [31] and therefore 16 studies were finally included in the quantitative meta-analysis [14, 16, 20, 23, 24, 30, 32–41], involving in total 3103 dystonia cases and 3628 healthy controls. In total 9 TOR1A SNPs have been investigated so far (rs1801968 and rs2296793 in ten studies, rs1182 and rs3842225 in nine, rs13283584, rs11787741 and rs13297609 in two and rs2287367 and rs1043186 in one study). One study revealed an association of a haplotype (constructed from rs2296793, rs1182 and rs3842225) with sporadic dystonia [36] while another one revealed a strong association of rs13283584 with idiopathic dystonia [38]. Association of rs1801968 with primary dystonia was reported in three studies [33, 39, 41] while another one revealed significant association only in cases with positive family history [32]. Association or a tendency for association of rs1182 was reported in three studies [37, 38, 40].

The majority of the studies were conducted in Chinese (n = 5) [20, 23, 34, 35, 41] and German (n = 4) [16, 30, 32, 38] populations. 10 studies reported inclusion of patients with positive family history of dystonia [20, 24, 32–35, 38–41] dystonia and in one study [41] was reported inclusion of participants positive for the ΔGAG TOR1A mutation. The characteristics of the included studies are summarized in **S1 Table**. Detailed information of the tested TOR1A SNPs is presented in **S2 Table**. There was no reported deviation from HWE in controls at any protocol. At another one, where two different populations were tested, we analyzed them separately in the meta-analysis [37].

### Tests of heterogeneity

Significant heterogeneity was revealed in the total dystonia group for a number of SNPs: rs1801968 (I^2^ = 53%, P_Q_ = 0.03 for dominant mode and I^2^ = 54.63%, P_Q_ = 0.002 for model free), rs2296793 (I^2^ = 42%, P_Q_<0.07 for recessive mode), rs1182 (I^2^ = 72%, P_Q_ = 0.00002 for dominant mode, I^2^ = 42%, P_Q_ = 0.08 for recessive mode and I^2^ = 81.98%, P_Q_<0.0001 for model free), rs13283584 (I^2^ = 79% and P_Q_ = 0.03 for both dominant and recessive modes and I^2^ = 65.72%, P_Q_ = 0.09 for model free), rs11787741 (I^2^ = 79%, P_Q_ = 0.03 for recessive mode). No significant heterogeneity was observed in the entire focal dystonia group and in the focal dystonia subgroups (cervical dystonia, blepharospasm and writer’s cramp). In case of considerable heterogeneity, the random-effects models were applied as previously described.

### Publication bias

**Fig 2:**
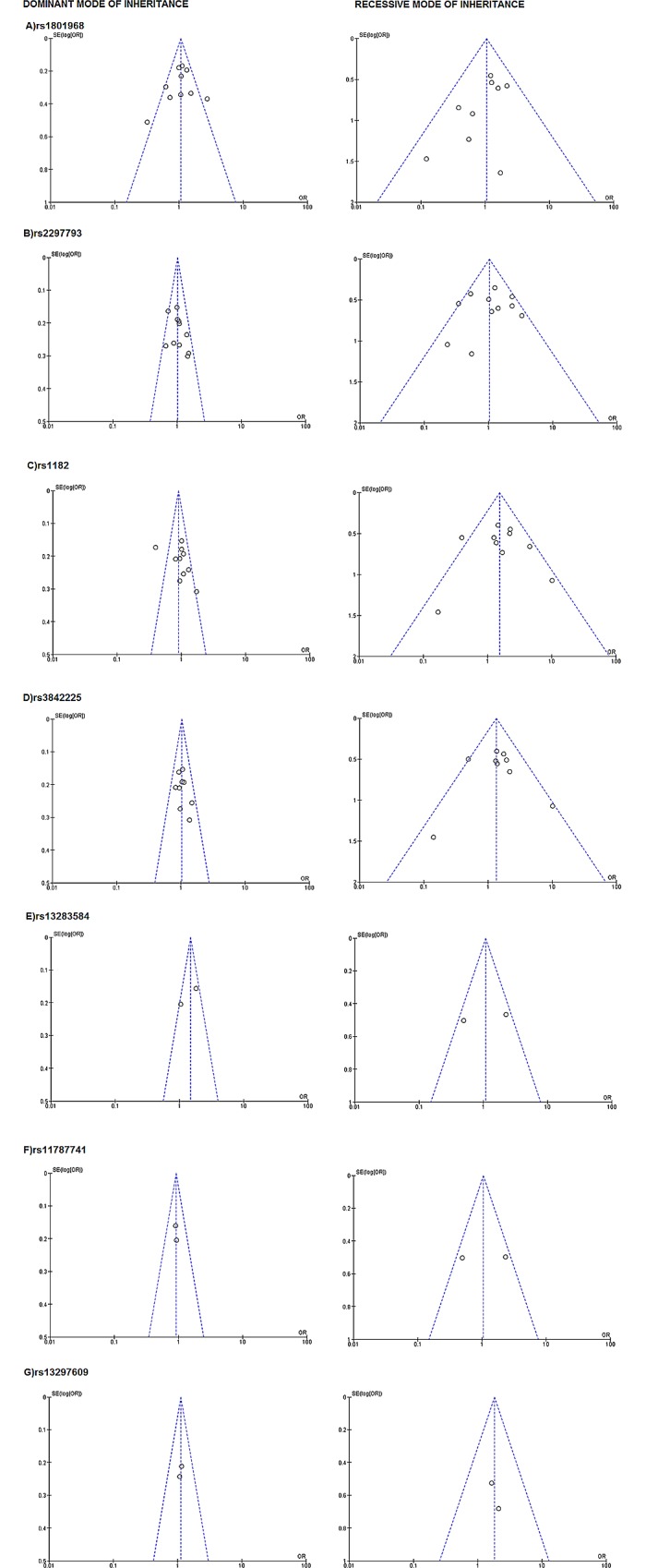
Funnel plot assessing evidence of publication bias in overall studies for TOR1A SNPs included in meta-analysis for overall dystonia group, in dominant and recessive modes of inheritance. SE, standard error; OR, odds ratio.

**Fig 3:**
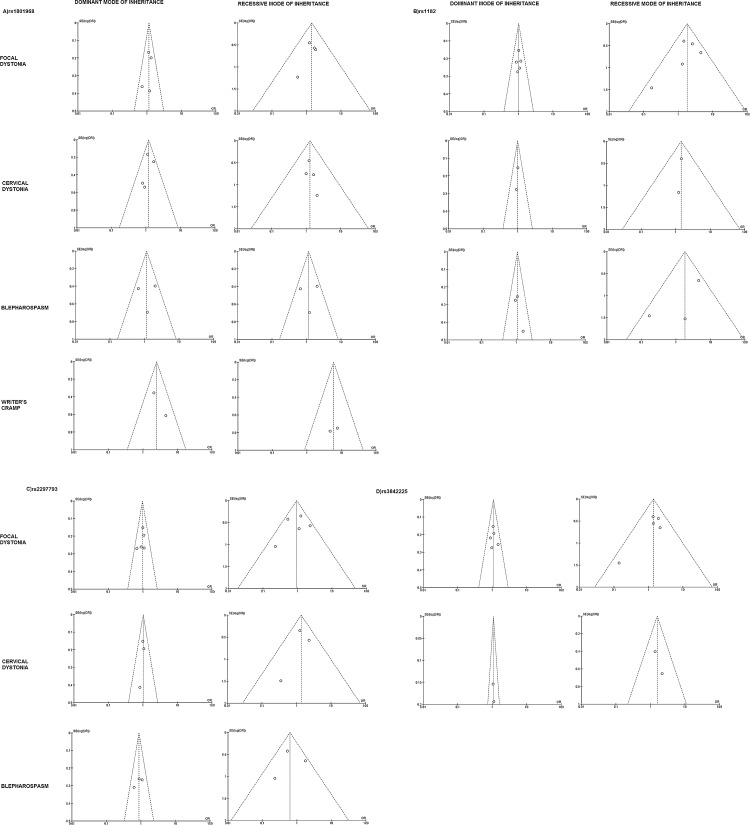
Funnel plot assessing evidence of publication bias in overall studies for TOR1A SNPs included in meta-analysis for entire focal dystonia group and focal dystonia subgroups (cervical dystonia, blepharospasm and writer’s cramp), in dominant and recessive modes of inheritance. SE, standard error; OR, odds ratio.

Funnel plots, which are presented in **Fig 2**for the overall dystonia group and in **Fig 3**for the focal dystonia group and focal dystonia subgroups, did not reveal any significant asymmetry for any tested SNP in the dominant or recessive modes. Results from Egger’s test (**S3 Appendix**) revealed no publication bias (P>0.10) in the dominant or recessive modes.

### Overall dystonia group analysis and subgroup analyses

**Fig 4:**
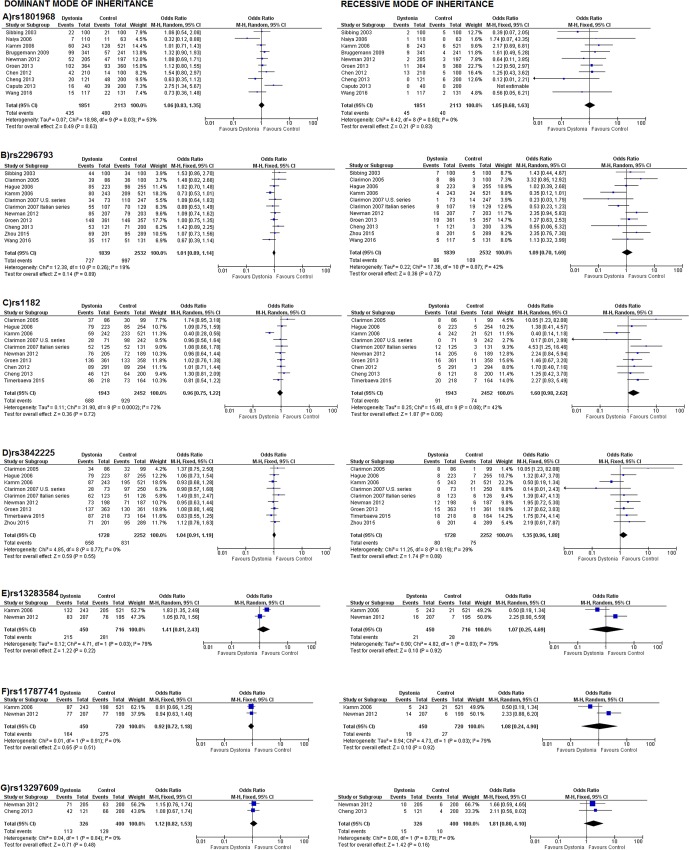
Odds ratios of dystonia associated with TOR1A polymorphisms in overall studies included in meta-analysis, in dominant (left) and recessive (right) modes of inheritance.

**Fig 5:**
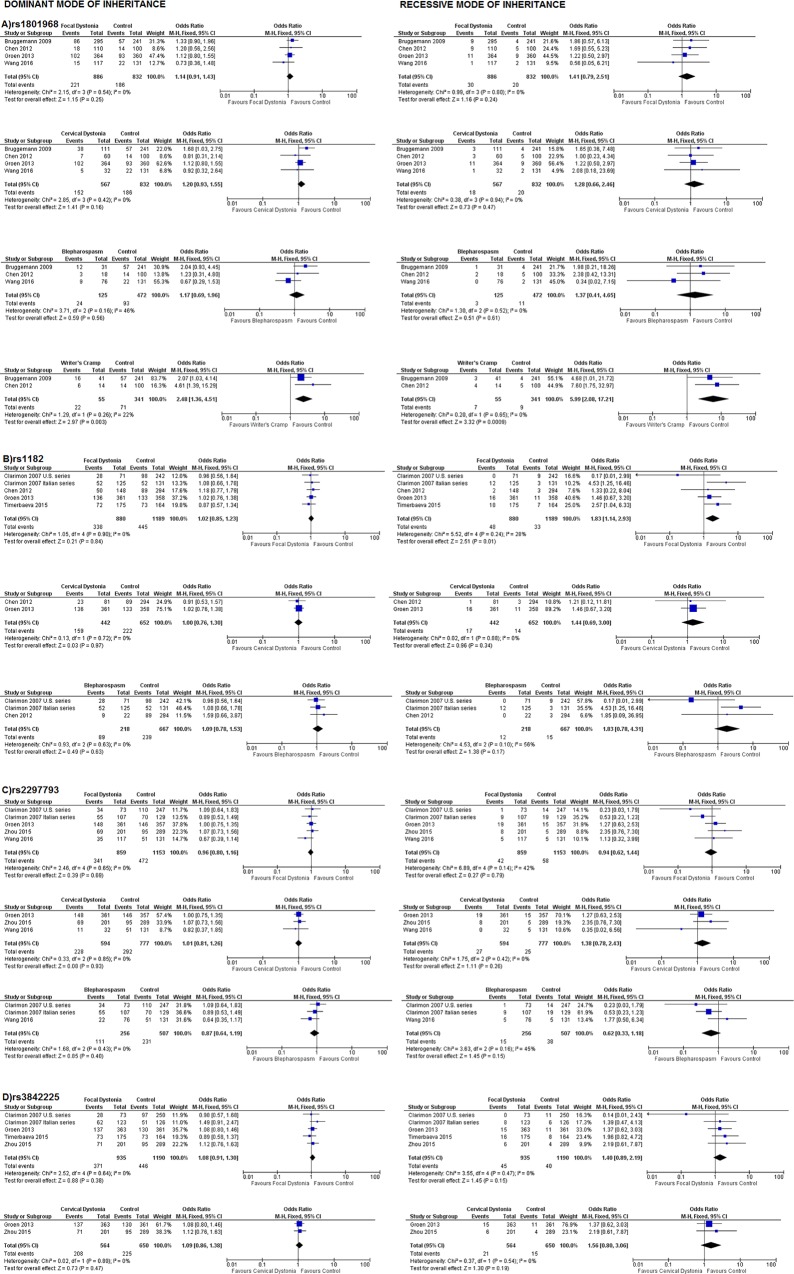
Odds ratios of entire focal dystonia group and focal dystonia subgroups (cervical dystonia, blepharospasm and writer’s cramp) associated with TOR1A polymorphisms in overall studies included in meta-analysis, in dominant (left) and recessive (right) modes of inheritance.

The main results of meta-analysis for all available SNPs in the overall dystonia group are presented as forest plots in **Fig 4**. A tendency towards association was found for rs1182 in the recessive inheritance mode [Odds Ratio, OR (95% confidence interval, C.I.): 1.60 (0.98–2.62)]. The main meta-analysis results for the entire focal dystonia group and the focal dystonia subgroups (cervical dystonia, blepharospasm and writer’s cramp) are shown in **Fig 5**. The respectively results after the meta-analysis with the model-fee approach and the genotypes frequencies, are showed at **Table 1**for the overall dystonia group and at **Table 2**for the focal dystonia group and focal dystonias’ subtypes. Overall, rs1182 was found to be associated with focal dystonia in recessive mode [OR (95%C.I.): 1.83 (1.14–2.93), P_z_ = 0.01]. Moreover, rs1801968 has found to be associated with writer’s cramp in both recessive and dominant modes [OR (95%C.I.): 5.99 (2.08–17.21), P_z_ = 0.00009] and [OR (95%C.I.): 2.48 (1.36–4.51), P_z_ = 0.003)] respectively and in model free-approach [ORG (95%C.I.): 2.58 (1.45–4.58)].

**Table 1:** Quantitate measures of genetic risk (individual study estimates and pooled effects), stratified by polymorphism of interest, for overall dystonia group, with the generalized odds ratio (OR_G_).

SNP	Author (Year)	Controls			Cases			Model-free	Model
rs1801968		wt	ht	mt	wt	Ht	Mt	OR_G_ (95%CI)	
	Siblling (2003)	79	16	5	78	20	2	1.02 (0.53–1.96)	
	Naiya (2006)	52	11	0	103	6	1	0.32 (0.12–0.85)	
	Kamm (2006)	393	122	6	183	54	6	1.02 (0.72–1.45)	
	Bruggemann (2009)	184	53	4	242	90	9	1.32 (0.91 1.91)	
	Newman(2012)	150	44	3	153	50	2	1.07 (0.69–1.68)	
	Groen (2013)	267	84	9	262	91	11	1.12 (0.81–1.54)	
	Chen (2012)	86	9	5	168	29	13	1.49 (0.79–2.82)	
	Cheng (2013)	152	42	6	101	20	0	0.63 (0.36–1.11)	
	Caputo (2013)	161	39	0	24	16	0	2.80 (1.37–5.70)	
	Wang (2016)	109	20	2	102	14	1	0.73 (0.36–1.47)	
	Pooled data	1633	440	40	1146	390	45	1.06 (0.83–1.35)	Random
	Heterogenity I^2^							54.63%	
	P_Q_							0.002	
**rs2296793**									
	Sibbing (2003)	66	29	5	56	37	7	1.49 (0.87–2.55)	
	Clarimon (2005)	64	33	3	47	31	8	1.55 (0.89–2.69)	
	Hague (2006)	159	87	9	138	77	8	1.02 (0.71–1.45)	
	Kamm (2006)	312	185	24	163	76	4	0.72(0.53–0.97)	
	Clarimon (2007) U.S.	137	96	14	39	33	1	1.00 (0.61–1.63)	
	Clarimon (2007) Italian	59	51	19	52	46	9	0.82 (0.52–1.29)	
	Newman(2012)	124	72	7	122	69	16	1.16(0.79–1.68)	
	Groen (2013)	211	131	15	213	129	19	1.02(0.77–1.35)	
	Cheng (2013)	129	68	3	68	52	1	1.39 (0.88–2.18)	
	Zhou (2015)	194	90	5	132	61	8	1.10 (0.76–1.59)	
	Wang (2016)	80	46	5	82	30	5	0.69 (0.42–1.16)	
	Pooled data	1535	888	109	1112	641	86	1.00 (0.89–1.14)	Fixed
	Heterogenity I^2^							30.89%	
	P_Q_							0.15	
**rs1182**									
	Clarimon (2005)	69	29	1	49	29	8	1.86(1.04–3.31)	
	Hague (2006)	169	80	5	144	73	6	1.09 (0.75–1.59)	
	Kamm (2006)	218	212	21	183	55	4	0.31 (0.22–0.44)	
	Clarimon (2007) U.S.	144	89	9	43	28	0	0.93 (0.55–1.56)	
	Clarimon (2007) Italian	79	49	3	73	40	12	1.19 (0.75–1.91)	
	Newman(2012)	117	66	6	129	62	14	1.02 (0.72–1.45)	
	Groen (2013)	225	122	11	225	120	16	1.04 0.78–1.39)	
	Chen (2012)	205	86	3	202	84	5	1.02 (0.72–1.45)	
	Cheng (2013)	136	56	8	75	40	6	1.29 (0.8–2.02)	
	Timerbaeva (2015)	91	66	7	132	66	20	0.91 (0.62–1.32)	
	Pooled data	1453	855	74	1255	597	91	0.97 (0.72–1.30)	Random
	Heterogenity I^2^							81.98%	
	P_Q_							<0.0001	
**rs3842225**									
	Clarimon (2005)	67	31	1	52	26	8	1.49 (0.84–2.66)	
	Hague (2006)	168	80	7	144	71	8	1.07 (0.74–1.54)	
	Kamm (2006)	326	174	21	156	82	5	0.91 (0.67–1.23)	
	Clarimon (2007) U.S.	153	86	11	45	28	0	0.94 (0.57–1.57)	
	Clarimon (2007) Italian	75	45	6	61	54	8	1.46 (0.91–2.33)	
	Newman(2012)	116	65	6	125	61	12	1.00 0.68–1.48)	
	Groen (2013)	231	119	11	226	122	15	1.09 (0.81–1.45)	
	Timerbaeva (2015)	91	65	8	131	69	18	0.89 (0.62 1.31)	
	Zhou (2015)	194	91	4	130	65	6	1.14 (0.78 1.65)	
	Pooled data	1421	756	75	1070	578	80	1.05 (0.93–1.20)	Fixed
	Heterogenity I^2^							00.0%	
	P_Q_							0.72	
**rs13283584**									
	Kamm (2006)	316	184	21	111	127	5	1.68 (1.26–2.24)	
	Newman(2012)	119	69	7	124	67	16	1.11 (0.76–1.62)	
	Pooled data	435	253	28	235	194	21	1.39 (0.93–2.08)	Random
	Heterogenity I^2^							65.72%	
	P_Q_							0.09	
**rs11787741**									
	Kamm (2006)	323	177	21	156	82	5	0.89 (0.66–1.20)	
	Newman(2012)	122	71	6	130	63	14	0.93 (0.68–1.46)	
	Pooled data	445	248	27	286	145	19	1.00 (0.73–1.18)	Fixed
	Heterogenity I^2^							0.00%	
	P_Q_							0.63	
**rs13297609**									
	Newman(2012)	137	57	6	134	61	10	1.17 (0.79–1.74)	
	Cheng (2013)	134	62	4	79	37	5	1.11 (0.70–1.76)	
	Pooled data	271	119	10	213	98	15	1.14 (0.85–1.54)	Fixed
	Heterogenity I^2^							0.00%	
	P_Q_							0.86	

SNP, single nucleotide polymorphism; wt, homozygotes for wild allele; ht, heterozygotes; mt, homozygotes for mutant allele; CI, confidence interval; OR_G_, generalized odds ratio.

**Table 2:** Quantitate measures of genetic risk (individual study estimates and pooled effects), stratified by polymorphism of interest, for focal dystonia group and its subtypes, with the generalized odds ratio (OR_G_).

Polymorphism/	Author (Year)	Controls			Cases			Model-free	Model
Phenotype									
rs1801968		wt	Ht	mt	wt	ht	mt	OR_G_ (95%CI)	
**Focal**									
**Dystonia**									
	Bruggemann (2009)	184	53	4	209	77	9	1.33 (0.91–1.95)	
	Chen (2012)	86	9	5	92	9	9	1.22 (0.59–2.53)	
	Groen (2013)	267	84	9	262	91	11	1.12 (0.81–1.54)	
	Wang (2016)	109	20	2	102	14	1	0.73 (0.36–1.47)	
	Pooled data	646	166	20	665	191	30	1.15 (0.92–1.43)	Fixed
	Heterogenity I^2^							0.00%	
	P_Q_							0.52	
**Cervical**									
**Dystonia**									
	Bruggemann (2009)	184	53	4	73	35	3	1.66 (1.03–2.69)	
	Chen (2012)	86	9	5	53	4	3	0.83 (0.32–2.12)	
	Groen (2013)	267	84	9	262	91	11	1.12 (0.81–1.54)	
	Wang (2016)	109	20	2	27	4	1	0.94 (0.33–2.66)	
	Pooled data	466	166	20	425	134	18	1.21 (0.94–1.55)	Fixed
	Heterogenity I^2^							0.00%	
	P_Q_							0.52	
**Blepharospasm**									
	Bruggemann (2009)	184	53	4	19	11	1	2.01 (0.94–4.27)	
	Chen (2012)	86	9	5	15	1	2	1.28 (0.34–4.83)	
	Wang (2016)	109	20	2	67	9	0	0.70 (0.31–1.58)	
	Pooled data	379	82	11	101	21	3	1.24 (0.74–2.06)	Fixed
	Heterogenity I^2^							42.26%	
	P_Q_							0.17	
**Writer’s**									
**cramp**									
	Bruggemann (2009)	184	53	4	25	13	3	2.10 (1.07–4.11)	
	Chen (2012)	86	9	5	8	2	4	4.52 (1.48–13.82)	
	Pooled data	270	62	9	33	15	7	**2.58 (1.45–4.58)**	Fixed
	Heterogenity I^2^							24.90%	
	P_Q_							0.24	
**rs1182**									
**Focal**									
**Dystonia**									
	Clarimon (2007) U.S.	144	89	9	43	28	0	0.93 (0.55–1.56)	
	Clarimon (2007) Italian	79	49	3	73	40	12	1.19 (0.75–1.91)	
	Chen (2012)	205	86	3	98	48	2	1.18 (0.78–1.78)	
	Groen (2013)	225	122	11	225	120	16	1.04 (0.78–1.39)	
	Timerbaeva (2015)	91	66	7	103	54	18	0.98 (0.66 1.46)	
	Pooled data	744	412	33	542	290	48	1.06 (0.89–1.26)	Fixed
	Heterogenity I^2^							0.00%	
	P_Q_							0.92	
**Cervical**									
**Dystonia**									
	Chen (2012)	205	86	3	58	22	1	0.92 (0.54–1.57)	
	Groen (2013)	225	122	11	225	120	16	1.04 (0.78–1.39)	
	Pooled data	430	208	14	283	142	17	1.01 (0.78–1.31)	Fixed
	Heterogenity I^2^							0.00%	
	P_Q_							0.69	
**Blepharospam**									
	Clarimon (2007) U.S.	144	89	9	43	28	0	0.93 (0.55–1.56)	
	Clarimon (2007) Italian	79	49	3	73	40	12	1.19 (0.75–1.91)	
	Chen (2012)	205	86	3	13	9	0	1.68 (0.71–3.96)	
	Pooled data	428	224	15	129	77	12	1.14 (0.83–1.57)	Fixed
	Heterogenity I^2^							0.00%	
	P_Q_							0.49	
**rs2296793**									
**Focal**									
**Dystonia**									
	Clarimon (2007) U.S.	137	96	14	39	33	1	1.00 (0.61–1.63)	
	Clarimon (2007) Italian	59	51	19	52	46	9	0.82 (0.52–1.29)	
	Groen (2013)	211	131	15	213	129	19	1.02 (0.77–1.35)	
	Zhou (2015)	194	90	5	132	61	8	1.10 (0.76–1.59)	
	Wang (2016)	80	46	5	82	30	5	0.70 (0.42–1.16)	
	Pooled data	681	414	58	518	299	42	0.96 (0.80–1.14)	Fixed
	Heterogenity I^2^							0.00%	
	P_Q_							0.61	
**Cervical**									
**Dystonia**									
	Groen (2013)	211	131	15	213	129	19	1.02 (0.77–1.35)	
	Zhou (2015)	194	90	5	132	61	8	1.10 (0.76–1.59)	
	Wang (2016)	80	46	5	21	11	0	0.84 (0.39–1.81)	
	Pooled data	485	267	25	366	201	27	1.03 (0.83–1.28)	Fixed
	Heterogenity I^2^							0.00%	
	P_Q_							0.82	
**Blepharospam**									
	Clarimon (2007) U.S.	137	96	14	39	33	1	1.00 (0.61–1.63)	
	Clarimon (2007) Italian	59	51	19	52	46	9	0.82 (0.52–1.29)	
	Wang (2016)	80	46	5	54	17	5	0.70 (0.39–1.25)	
	Pooled data	276	193	38	145	96	15	0.85 (0.63–1.13)	Fixed
	Heterogenity I^2^							0.00%	
	P_Q_							0.65	
**rs3842225**									
**Focal**									
**Dystonia**									
	Clarimon (2007) U.S.	153	86	11	45	28	0	0.94 (0.57–1.57)	
	Clarimon (2007) Italian	75	45	6	61	54	8	1.46 (0.91–2.33)	
	Groen (2013)	231	119	11	226	122	15	1.09 (0.81–1.45)	
	Timerbaeva (2015)	91	65	8	102	57	16	0.97 (0.75–1.45)	
	Zhou (2015)	194	91	4	130	65	6	1.14 (0.78–1.65)	
	Pooled data	744	406	40	564	326	45	1.10 (0.93–1.31)	Fixed
	Heterogenity I^2^							0.00%	
	P_Q_							0.71	
**Cervical**									
**Dystonia**									
	Groen (2013)	231	119	11	226	122	15	1.09 (0.81–145)	Fixed
	Zhou (2015)	194	91	4	130	65	6	1.14 (0.78–1.65)	
	Pooled data	425	210	15	356	187	21	1.11 (0.88–1.39)	
	Heterogenity I^2^							0.00%	
	P_Q_							0.86	

SNP, single nucleotide polymorphism; wt, homozygotes for wild allele; ht, heterozygotes; mt, homozygotes for mutant allele; CI, confidence interval; OR_G_, generalized odds ratio; Statistical significant OR_G_s of the pooled data are given in bold.

## Discussion

In the present meta-analysis, that included a relatively large number of participants, we investigated the effect of TOR1A gene SNPs on the risk of dystonia, as well as on the risk of focal dystonia and its subtypes (cervical dystonia, blepharospasm and writer’s cramp). Our study detected a significant influence of a specific variant of TOR1A gene, rs1182, on the risk of focal dystonia. It also showed and that there is a strong association between rs1801968 and the development of task specific writer’s cramp focal dystonia. To the best of our knowledge, this is the first meta-analysis examining the effect of TOR1A gene polymorphisms on the above-mentioned disorders.

Quite a few polymorphisms across TOR1A gene have been examined for possible association with dystonia, but the results from candidate gene association studies (CGASs) remain conflicting [20, 23]. Additionally, in the first meta-analysis and in a pooled analysis regarding TOR1A SNPs and dystonia no significant association was revealed [14, 23]. However, in a second meta-analysis a marginal statistical significance was revealed for rs1801968 only in a subgroup of patients with primary dystonia that also had a positive family history [24]. The lack of replication and the inconsistency of the results among CGASs, meta- and pooled- analyses about TOR1A gene and dystonia could be attributed to a number of reasons. Small sample sizes, low statistical power to detect associations and different ethnic backgrounds may have contributed to these discrepancies [24, 31]. Our study pooled patients from different ethnic groups. Specifically, rs1182 exhibited a significant effect in a cohort with focal dystonia (n = 880) consisting of a variety of nationalities (Caucasians, Africans, Americans, Hispanic, Italians, Chinese, Dutch and Slavs). Also, rs1801968 was found to be associated with writer’s cramp in a cohort (n = 55) of German and Chinese patients. The large clinical phenotypic spectrum of dystonia and the fact that a dystonia patient can be assign in more than one phenotypic group [7, 14] could be an additional factor leading to conflicting results [14, 23]. In our study rs1801968 was found to be associated only with writer’s cramp sub-phenotype and not with the entire focal dystonia phenotype. Moreover, the variability in the penetrance of mutations, the epistasis phenomenon and the interaction between genetic and environmental factors could be responsible for the discordance of the results [1, 24, 42].

The ΔGAG human variant of TorsinA was the first pathogenic, disease-causing mutation identified in early onset, generalized dystonia [43]. Thereafter, functional characterization of the growing number of the identified TOR1A variants in dystonia patients signifies the importance of the whole genetic variability across TOR1A gene [44]. Rs1801968 (D216H) is a functional missence coding polymorphism in exon 4 of TOR1A gene, which replaces the amino acid aspartic acid (D) with histidine (H) at residue 216 [45]. In a cell culture study, this amino acid substitution predisposes to the formation of inclusions similar to those formed by ΔGAG mutation [45]. However, the co-existence of 216H allele and ΔGAG mutation reduced the tendency for inclusions’ formation [45]. This finding suggests that rs1801968 may have a protective role against the effect of ΔGAG mutation and dystonia [45–47]. On the contrary, 216D allele in cis together with ΔGAG, may predispose for developing dystonia [46, 47]. Cheng et al. reported that 216D allele was more abundant in patients with early-onset primary dystonia, while 216H allele was more frequent in the control group [41]. Furthermore, they observed that all the ΔGAG carriers, who also carried the 216D allele, were dystonic [41]. However, the great variance in the results from studies regarding the impact of rs18019168 on dystonia [23], suggests that additional factors influence the phenotypic manifestation of this polymorphism.

Rs1182, that also reached significance in our study, is located in the 3’ untranslated region (3’-UTR) of exon 5 of TOR1A gene. However, there is no known functional effect of rs1182 on expression and function of TOR1A [48] so, the precise functional role of rs1182 on primary dystonia remains unclear. In addition to the effect of rs1182 on the risk of dystonia [36–38, 40], minor rs1182 allele may also represent a genetic factor influencing the spread of blepharospasm to adjacent body regions [49]. The role of another SNP, in particular rs3842225 polymorphism, that is also located in TOR1A 3’-UTR region of exon has also been examined in dystonia patients, but with ambiguous results [20]. There is only some indications that del allele of rs3842225 in cis with D216 allele of rs1801968 may be associated with reduced risk of dystonia [31, 47]. Furthermore, Clarimon et al. conducted a population-based study and described the association between dystonia and T2 haplotype which is consists of the minor alleles of rs1182, rs3842225 and rs2296793 [36]. No such association was revealed when each one of these three SNPs was examined separately [36]. Therefore, it is possible that SNPs within 3’-UTR of exon 5 represent an additional functional genetic locus of TOR1A, though it may be under synergic action with other TOR1A genetic variants.

Both variants emerged in our analysis may have some functional consequences. In particular, rs1182 is located in the UTR-3 that may affect transcription of TOR1A gene, whereas rs1801968 represents a missense mutation and is characterized as pathogenic in NCBI. In addition, an almost consistent effect across varied populations is also supportive of the significant role of both polymorphisms and by extension TOR1A gene. Moreover, the heterogeneity of effect size across studies it remained within acceptable limits (I^2^: 0–28%). However, it is not known whether these associations are causal without supportive functional analyses.

There are certain limitations in the present meta-analysis that must be acknowledged. [40]. Firstly, we included subjects regardless of the ΔGAG mutation status, the diagnostic methodology, the HWE values and the presence of positive family history or decent. We cannot also exclude a possible classification bias regarding the assignment of participants in dystonia phenotypes, as the majority of studies were performed before the dystonias’ classification consensus update.

In conclusion, our meta-analysis revealed a possible influence of rs1182 TOR1A SNP on the risk of focal dystonia and of rs1801968 on the risk of writer’s cramp. Further large-scale collaborative studies in different ethnic groups, with larger samples and with prospective and gene-environment interaction design are of great necessity in order to elucidate the role of TOR1A gene in focal dystonia. The investigation of additional genetic factors that predispose to dystonia may provide physicians with personalized tools for diagnosis, classification or treatment response.

## Supporting Information

S1 Appendix The complete search algorithm. (PDF)

S2 Appendix PRISMA 2009 Checklist. (PDF)

S3 Appendix Results from Egger’s test. (PDF)

S1 Table Characteristics of the studies included in the meta-analysis. (DOCX)

S2 Table Characteristics of TOR1A SNPs that examined in the current meta-analysis. (DOCX)

## References

[1] Charlesworth G, Bhatia KP, Wood NW. The genetics of dystonia: new twists in an old tale. Brain: a journal of neurology. 2013;136(Pt 7):2017–37. Epub 2013/06/19. PubMed Central PMCID: PMCPmc3692036. 23775978 10.1093/brain/awt138 PMC3692036

[2] Standaert DG. Update on the pathology of dystonia. Neurobiology of disease. 2011;42(2):148–51. Epub 2011/01/12. PubMed Central PMCID: PMCPmc3073692. 10.1016/j.nbd.2011.01.012 21220015 PMC3073692

[3] Quartarone A, Morgante F, Sant'angelo A, Rizzo V, Bagnato S, Terranova C, et al Abnormal plasticity of sensorimotor circuits extends beyond the affected body part in focal dystonia. Journal of neurology, neurosurgery, and psychiatry. 2008;79(9):985–90. Epub 2007/07/20. 10.1136/jnnp.2007.121632 17634214

[4] Breakefield XO, Blood AJ, Li Y, Hallett M, Hanson PI, Standaert DG. The pathophysiological basis of dystonias. Nature reviews Neuroscience. 2008;9(3):222–34. Epub 2008/02/21. 10.1038/nrn2337 18285800

[5] Teo JT, van de Warrenburg BP, Schneider SA, Rothwell JC, Bhatia KP. Neurophysiological evidence for cerebellar dysfunction in primary focal dystonia. Journal of neurology, neurosurgery, and psychiatry. 2009;80(1):80–3. Epub 2008/12/19. 10.1136/jnnp.2008.144626 19091711

[6] Argyelan M, Carbon M, Niethammer M, Ulug AM, Voss HU, Bressman SB, et al Cerebellothalamocortical connectivity regulates penetrance in dystonia. The Journal of neuroscience: the official journal of the Society for Neuroscience. 2009;29(31):9740–7. Epub 2009/08/07. PubMed Central PMCID: PMCPmc2745646. 19657027 10.1523/JNEUROSCI.2300-09.2009 PMC2745646

[7] Albanese A, Bhatia K, Bressman SB, Delong MR, Fahn S, Fung VS, et al Phenomenology and classification of dystonia: a consensus update. Movement disorders: official journal of the Movement Disorder Society. 2013;28(7):863–73. Epub 2013/05/08. PubMed Central PMCID: PMCPmc3729880. 23649720 10.1002/mds.25475 PMC3729880

[8] Hettich J, Ryan SD, de Souza ON, Saraiva Macedo Timmers LF, Tsai S, Atai NA, et al Biochemical and cellular analysis of human variants of the DYT1 dystonia protein, TorsinA/TOR1 A. Human mutation. 2014;35(9):1101–13. Epub 2014/06/17. PubMed Central PMCID: PMCPmc4134760. 10.1002/humu.22602 24930953 PMC4134760

[9] Ozelius LJ, Hewett JW, Page CE, Bressman SB, Kramer PL, Shalish C, et al The early-onset torsion dystonia gene (DYT1) encodes an ATP-binding protein. Nature genetics. 1997;17(1):40–8. Epub 1997/09/01. 10.1038/ng0997-40 9288096

[10] Balint B, Bhatia KP. Isolated and combined dystonia syndromes—an update on new genes and their phenotypes. European journal of neurology. 2015;22(4):610–7. Epub 2015/02/04. 10.1111/ene.12650 25643588

[11] Brancati F, Valente EM, Castori M, Vanacore N, Sessa M, Galardi G, et al Role of the dopamine D5 receptor (DRD5) as a susceptibility gene for cervical dystonia. Journal of neurology, neurosurgery, and psychiatry. 2003;74(5):665–6. Epub 2003/04/18. PubMed Central PMCID: PMCPmc1738453. 10.1136/jnnp.74.5.665 12700316 PMC1738453

[12] Misbahuddin A, Placzek MR, Chaudhuri KR, Wood NW, Bhatia KP, Warner TT. A polymorphism in the dopamine receptor DRD5 is associated with blepharospasm. Neurology. 2002;58(1):124–6. Epub 2002/01/10. 11781417 10.1212/wnl.58.1.124

[13] Misbahuddin A, Placzek MR, Warner TT. Focal dystonia is associated with a polymorphism of the dopamine D5 receptor gene. Advances in neurology. 2004;94:143–6. Epub 2003/09/27. 14509667

[14] Newman JR, Sutherland GT, Boyle RS, Limberg N, Blum S, O'Sullivan JD, et al Common polymorphisms in dystonia-linked genes and susceptibility to the sporadic primary dystonias. Parkinsonism & related disorders. 2012;18(4):351–7. Epub 2011/12/17. 22172551 10.1016/j.parkreldis.2011.11.024

[15] Placzek MR, Misbahuddin A, Chaudhuri KR, Wood NW, Bhatia KP, Warner TT. Cervical dystonia is associated with a polymorphism in the dopamine (D5) receptor gene. Journal of neurology, neurosurgery, and psychiatry. 2001;71(2):262–4. Epub 2001/07/19. PubMed Central PMCID: PMCPmc1737490. 10.1136/jnnp.71.2.262 11459908 PMC1737490

[16] Sibbing D, Asmus F, Konig IR, Tezenas du Montcel S, Vidailhet M, Sangla S, et al Candidate gene studies in focal dystonia. Neurology. 2003;61(8):1097–101. Epub 2003/10/29. 14581671 10.1212/01.wnl.0000090560.20641.ab

[17] Chen Y, Song W, Yang J, Chen K, Huang R, Zhao B, et al Association of the Val66Met polymorphism of the BDNF gene with primary cranial-cervical dystonia patients from South-west China. Parkinsonism & related disorders. 2013;19(11):1043–5. Epub 2013/07/03. 23816543 10.1016/j.parkreldis.2013.06.004

[18] Cramer SC, Sampat A, Haske-Palomino M, Nguyen S, Procaccio V, Hermanowicz N. Increased prevalence of val(66)met BDNF genotype among subjects with cervical dystonia. Neuroscience letters. 2010;468(1):42–5. Epub 2009/10/28. PubMed Central PMCID: PMCPmc3270368. 10.1016/j.neulet.2009.10.059 19857550 PMC3270368

[19] Sako W, Murakami N, Izumi Y, Kaji R. Val66Met polymorphism of brain-derived neurotrophic factor is associated with idiopathic dystonia. Journal of clinical neuroscience: official journal of the Neurosurgical Society of Australasia. 2015;22(3):575–7. Epub 2014/12/20. 25523127 10.1016/j.jocn.2014.08.014

[20] Zhou Q, Chen Y, Yang J, Cao B, Wei Q, Ou R, et al Association analysis of TOR1A polymorphisms rs2296793 and rs3842225 in a Chinese population with cervical dystonia. Neuroscience letters. 2015;612:185–8. Epub 2015/12/26. 10.1016/j.neulet.2015.12.030 26704435

[21] Xiromerisiou G, Dardiotis E, Tsironi EE, Hadjigeorgiou G, Ralli S, Kara E, et al THAP1 mutations in a Greek primary blepharospasm series. Parkinsonism & related disorders. 2013;19(3):404–5. Epub 2012/10/06. 23036512 10.1016/j.parkreldis.2012.08.015

[22] Matsumoto S, Nishimura M, Sakamoto T, Asanuma K, Izumi Y, Shibasaki H, et al Modulation of the onset age in primary dystonia by APOE genotype. Neurology. 2003;60(12):2003–5. Epub 2003/06/25. 12821754 10.1212/01.wnl.0000068161.38412.1f

[23] Wang L, Duan C, Gao Y, Xu W, Ding J, Liu VT, et al Lack of association between TOR1A and THAP1 mutations and sporadic adult-onset primary focal dystonia in a Chinese population. Clinical neurology and neurosurgery. 2016;142:26–30. Epub 2016/01/25. 10.1016/j.clineuro.2016.01.018 26803725

[24] Groen JL, Ritz K, Tanck MW, van de Warrenburg BP, van Hilten JJ, Aramideh M, et al Is TOR1A a risk factor in adult-onset primary torsion dystonia? Movement disorders: official journal of the Movement Disorder Society. 2013;28(6):827–31. Epub 2013/03/06. 23460578 10.1002/mds.25381

[25] Zintzaras E. The generalized odds ratio as a measure of genetic risk effect in the analysis and meta-analysis of association studies. Statistical applications in genetics and molecular biology. 2010;9:Article21. Epub 2010/07/06. 10.2202/1544-6115.1542 20597847

[26] Zintzaras E. The power of generalized odds ratio in assessing association in genetic studies with known mode of inheritance. Journal of Applied Statistics. 2012;39(12):2569–81.

[27] Mantel N, Haenszel W. Statistical aspects of the analysis of data from retrospective studies of disease. Journal of the National Cancer Institute. 1959;22(4):719–48. Epub 1959/04/01. 13655060

[28] DerSimonian R, Laird N. Meta-analysis in clinical trials. Controlled clinical trials. 1986;7(3):177–88. Epub 1986/09/01. 3802833 10.1016/0197-2456(86)90046-2

[29] Egger M, Davey Smith G, Schneider M, Minder C. Bias in meta-analysis detected by a simple, graphical test. BMJ (Clinical research ed). 1997;315(7109):629–34. Epub 1997/10/06. PubMed Central PMCID: PMCPmc2127453. 10.1136/bmj.315.7109.629 PMC2127453 9310563

[30] Hague S, Klaffke S, Clarimon J, Hemmer B, Singleton A, Kupsch A, et al Lack of association with TorsinA haplotype in German patients with sporadic dystonia. Neurology. 2006;66(6):951–2. Epub 2006/03/29. 10.1212/01.wnl.0000203344.43342.18 16567727

[31] Sharma N, Franco RA Jr., Kuster JK, Mitchell AA, Fuchs T, Saunders-Pullman R, et al Genetic evidence for an association of the TOR1A locus with segmental/focal dystonia. Movement disorders: official journal of the Movement Disorder Society. 2010;25(13):2183–7. Epub 2010/07/30. PubMed Central PMCID: PMCPmc3095887. 20669276 10.1002/mds.23225 PMC3095887

[32] Bruggemann N, Kock N, Lohmann K, Konig IR, Rakovic A, Hagenah J, et al The D216H variant in the DYT1 gene: a susceptibility factor for dystonia in familial cases? Neurology. 2009;72(16):1441–3. Epub 2009/04/22. 10.1212/WNL.0b013e3181a1861e 19380705

[33] Caputo M, Irisarri M, Perandones C, Alechine E, Pellene LA, Roca CU, et al Analysis of D216H polymorphism in Argentinean patients with primary dystonia. Journal of neurogenetics. 2013;27(1–2):16–8. Epub 2013/02/15. 10.3109/01677063.2012.761697 23405979

[34] Chen Y, Burgunder JM, Song W, Huang R, Shang HF. Assessment of D216H DYT1 polymorphism in a Chinese primary dystonia patient cohort. European journal of neurology. 2012;19(6):924–6. Epub 2011/11/08. 10.1111/j.1468-1331.2011.03582.x 22054283

[35] Chen Y, Chen K, Burgunder JM, Song W, Huang R, Zhao B, et al Association of rs1182 polymorphism of the DYT1 gene with primary dystonia in Chinese population. Journal of the neurological sciences. 2012;323(1–2):228–31. Epub 2012/10/13. 10.1016/j.jns.2012.09.025 23058565

[36] Clarimon J, Asgeirsson H, Singleton A, Jakobsson F, Hjaltason H, Hardy J, et al Torsin A haplotype predisposes to idiopathic dystonia. Annals of neurology. 2005;57(5):765–7. Epub 2005/04/27. 10.1002/ana.20485 15852391

[37] Clarimon J, Brancati F, Peckham E, Valente EM, Dallapiccola B, Abruzzese G, et al Assessing the role of DRD5 and DYT1 in two different case-control series with primary blepharospasm. Movement disorders: official journal of the Movement Disorder Society. 2007;22(2):162–6. Epub 2006/11/30. 17133500 10.1002/mds.21182

[38] Kamm C, Asmus F, Mueller J, Mayer P, Sharma M, Muller UJ, et al Strong genetic evidence for association of TOR1A/TOR1B with idiopathic dystonia. Neurology. 2006;67(10):1857–9. Epub 2006/11/30. 10.1212/01.wnl.0000244423.63406.17 17130424

[39] Naiya T, Biswas A, Neogi R, Datta S, Misra AK, Das SK, et al Clinical characterization and evaluation of DYT1 gene in Indian primary dystonia patients. Acta neurologica Scandinavica. 2006;114(3):210–5. Epub 2006/08/17. 10.1111/j.1600-0404.2006.00663.x 16911351

[40] Timerbaeva SL, Abramycheva NY, Rebrova OY, Illarioshkin SN. TOR1A polymorphisms in a Russian cohort with primary focal/segmental dystonia. The International journal of neuroscience. 2015;125(9):671–7. Epub 2014/09/10. 10.3109/00207454.2014.962653 25203860

[41] Cheng FB, Wan XH, Zhang Y, Miao J, Sun Y, Sun YB, et al TOR1A sequence variants and the association with early-onset primary dystonia in the Chinese Han population. Parkinsonism & related disorders. 2013;19(3):399–401. Epub 2012/10/31. 23107556 10.1016/j.parkreldis.2012.08.013

[42] Defazio G, Berardelli A, Hallett M. Do primary adult-onset focal dystonias share aetiological factors? Brain. 2007;130(Pt 5):1183–93. Epub 2007/01/24. 10.1093/brain/awl355 17242025

[43] Klein C. Genetics in dystonia. Parkinsonism & related disorders. 2014;20 Suppl 1:S137–42. Epub 2013/11/23. 24262166 10.1016/S1353-8020(13)70033-6

[44] Dobricic V, Kresojevic N, Zarkovic M, Tomic A, Marjanovic A, Westenberger A, et al Phenotype of non-c.907_909delGAG mutations in TOR1A: DYT1 dystonia revisited. Parkinsonism & related disorders. 2015;21(10):1256–9. Epub 2015/08/25. 26297380 10.1016/j.parkreldis.2015.08.001

[45] Kock N, Naismith TV, Boston HE, Ozelius LJ, Corey DP, Breakefield XO, et al Effects of genetic variations in the dystonia protein torsinA: identification of polymorphism at residue 216 as protein modifier. Human molecular genetics. 2006;15(8):1355–64. Epub 2006/03/16. 10.1093/hmg/ddl055 16537570

[46] Kamm C, Fischer H, Garavaglia B, Kullmann S, Sharma M, Schrader C, et al Susceptibility to DYT1 dystonia in European patients is modified by the D216H polymorphism. Neurology. 2008;70(23):2261–2. Epub 2008/06/04. 10.1212/01.wnl.0000313838.05734.8a 18519876

[47] Risch NJ, Bressman SB, Senthil G, Ozelius LJ. Intragenic Cis and Trans modification of genetic susceptibility in DYT1 torsion dystonia. American journal of human genetics. 2007;80(6):1188–93. Epub 2007/05/16. PubMed Central PMCID: PMCPmc1867106. 10.1086/518427 17503336 PMC1867106

[48] Lohmann K, Klein C. Genetics of dystonia: what's known? What's new? What's next? Movement disorders: official journal of the Movement Disorder Society. 2013;28(7):899–905. Epub 2013/07/31. 23893446 10.1002/mds.25536

[49] Defazio G, Matarin M, Peckham EL, Martino D, Valente EM, Singleton A, et al The TOR1A polymorphism rs1182 and the risk of spread in primary blepharospasm. Movement disorders: official journal of the Movement Disorder Society. 2009;24(4):613–6. Epub 2009/02/10. PubMed Central PMCID: PMCPmc4167593. 19202559 10.1002/mds.22471 PMC4167593

