# Supplementary material for: The Role of TOR1A Polymorphisms in Dystonia: A Systematic Review and Meta-Analysis
Source: PLoS One. 2017 Jan 12;12(1):e0169934. doi: 10.1371/journal.pone.0169934 (PMC5231385; doi:10.1371/journal.pone.0169934)
Supplement: S1 Appendix — (PDF) [file pone.0169934.s001.pdf]

("dystonic disorders"[MeSH Terms] OR ("dystonic"[All Fields] AND "disorders"[All Fields]) OR "dystonic disorders"[All Fields] OR "dystonia"[All Fields] OR "dystonia"[MeSH Terms]) AND tor1a[All Fields] AND ("polymorphism, genetic"[MeSH Terms] OR ("polymorphism"[All Fields] AND "genetic"[All Fields]) OR "genetic polymorphism"[All Fields] OR "polymorphism"[All Fields])
