# Supplementary material for: The Role of TOR1A Polymorphisms in Dystonia: A Systematic Review and Meta-Analysis
Source: PLoS One. 2017 Jan 12;12(1):e0169934. doi: 10.1371/journal.pone.0169934 (PMC5231385; doi:10.1371/journal.pone.0169934)
Supplement: S3 Appendix — (PDF) [file pone.0169934.s003.pdf]

P-values for Egger's tests.

**Phenotype/ SNP**	**Dystonia**		**Focal Dystonia**		**Cervical Dystonia**		**Blepharospasm**		**Writer's Cramp**	
	Dominant	Recessive	Dominant	Recessive	Dominant	Recessive	Dominant	Recessive	Dominant	Recessive
rs1801968	0.535	0.205	0.519	0.506	0.701	0.410	0.932	0.888	NA	NA
rs2296793	0.207	0.671	0.404	0.656	0.470	0.797	0.338	0.943		
rs1182	0.272	0.584	0.904	0.828	NA	NA	0.314	NA		
rs3842225	0.796	0.422	0.820	0.804	NA	NA				
rs3842225	0.796	0.422								
rs13283584	NA	NA								
rs11787741	NA	NA								
rs13297609	NA	NA								

SNP, single nucleotide polymorphism; NA, Non-available.
