# Supplementary material for: The Role of TOR1A Polymorphisms in Dystonia: A Systematic Review and Meta-Analysis
Source: PLoS One. 2017 Jan 12;12(1):e0169934. doi: 10.1371/journal.pone.0169934 (PMC5231385; doi:10.1371/journal.pone.0169934)
Supplement: S1 Table — (DOCX) [file pone.0169934.s004.docx]

***S1 Table:*** *Characteristics of the studies included in the meta-analysis.*

|  | | | | | | **Cases** | | | **Controls** | | |  |
| --- | --- | --- | --- | --- | --- | --- | --- | --- | --- | --- | --- | --- |
| **Author^[ref.]^** | **Population** | **Tested SNPs** | **Participants with ΔGAG mutation** | **Cases with positive family history** | **Examined dystonias’ phenotypes** | **Mean age±SD/** | **n** | **Male/** | **Mean Age±SD** | **n** | **Male/** | **Main Results** |
| **(Year)** |  |  |  |  |  |  |  | **Female** |  |  | **Female** |  |
|  |  |  |  |  |  | **Age of onset±SD** |  |  |  |  |  |  |
| Sibbing^[16]^ | German | rs1801968, rs2296793 | No | - | primary focal dystonia (blepharospasm, Meige syndrome, torticollis, writer's cramp) | 55.0±15.6/ | 100 | 36/ | 54.7±15.7 | 100 | 36/ | Negative |
| (2003) |  |  |  |  |  | 44.5±16.3 |  | 64 |  |  | 64 |  |
| Clarimon^[36]^ | Icelandic | rs2296793, rs1182, rs3842225 | No | - | primary idiopathic dystonia | NA/ | 86 | NA/ | NA | 100 | NA/ | Association between A-T-del haplotype and sporadic dystonia, [p=0.005, OR=1.72, 95%CI=(1.02–2.91)] |
| (2005) |  |  |  |  |  | NA |  | NA |  |  | NA |  |
| Hague^[30]^ | German | rs2296793, rs1182, rs3842225 | - | - | sporadic (torticollis, blepharospasm, writer’s cramp, dystonia of one limb, multifocal, segmental dystonia) | 59.80±12.74/ | 223 | 84/ | 47.95±15.97 | 255 | 118/ | Negative |
| (2006) |  |  |  |  |  | NA |  | 139 |  |  | 137 |  |
| Kamm^[38]^ | Southern German/ | rs1801968, rs2296793, rs1182, rs3842225, rs13283584, rs11787741 | No | Yes | sporadic primary (torticollis, cranial, writer’s cramp, spasmodic dysphonia, segmental dystonia) | NA/ | 243 | 102/ | NA | 521 | 306/ | Strong association for rs1182 (p=0.00001) and rs13283584 (p=0.000008) at genotypic model with dystonia |
| (2006) |  |  |  |  |  | NA |  | 141 |  |  | 215 |  |
|  | Austrian |  |  |  |  |  |  |  |  |  |  |  |
| Naiya^[39]^ | Indian | rs1801968 | No | Yes | primary dystonia (writer’s cramp, cervical dystonia, other focal, segmental generalized, other) | NA/ | 110 | NA/ | 60±12 | 63 | NA/ | Association of rs1801968 with primary dystonia at genotypic model (p≤0.05) and at allelic model [p≤0.05, OR=2.535, 95%CI=(0.9915-6.4799)] |
| (2006) |  |  |  |  |  | NA |  | NA |  |  | NA |  |
| Clarimon^[37]^ | US (caucasian, african-america, hispanic) | rs2296793, rs1182, rs3842225 | - | - | primary blepharospasm | 63.8/ | 75 | NA/ | 67 | 251 | NA/ | Negative |
| (2007) |  |  |  |  |  | NA |  | 74% |  |  | 65.8% |  |
|  | Italian |  |  |  |  | 69.2/ | 130 | NA/ | NA | 130 | NA/ | An overrepresentation of the rs1182 TT genotype was found in patients compared to controls (p=0.041; OR= 4.53, 95%CI=1.25–16.47). The statistical significance did not survive after corrections for multiple comparisons |
|  |  |  |  |  |  | NA |  | 67% |  |  | NA |  |
| Bruggemann^[32]^ | German | rs1801968, rs3842225 | No | Yes | blepharospasm, cervical dystonia, writer’s cramp, musician’s dystonia, segmental dystonia | 51.9/ | 341 | 48.7%/ | 59.1 | 241 | 51.1%/ | Higher frequency of the H216 variant of the rs1801968 in familial dystonia cases compared to controls was detected at Cochrane-Armitage trend test (exact two-sided p=0.011). This difference was significant in the cervical dystonia, writer’s cramp, and blepharospasm subgroups and maintained after Bonferroni-Holm correction for multiple comparison (p =0.022) |
| (2009) |  |  |  |  |  | 40.1 |  | 51.3% |  |  | 48.9% |  |
| Chen^[34]^ | Southwest Chinese | rs1801968 | No | Yes | primary (blepharospasm, cervical, writer’s cramp, other focal, Meige syndrome, other segmental, generalized, multifocal) | NA/ | 210 | 89/ | NA | 100 | NA/ | Negative. The frequency of allele C in writer’s cramp was higher than that in other types of focal dystonia (p= 0.0006517) |
| (2012) |  |  |  |  |  | 36.90±19.01 |  | 121 |  |  | NA |  |
| Chen^[35]^ | Chinese | rs1182 | No | Yes | primary “pure” (blepharospasm, cervical, writer’s cramp, other focal, Meige syndrome, cranial-cervical, other segmental, generalized, multiple) | NA/ | 291 | 125/ | 37.35±19.00 | 294 | NA/ | Negative |
| (2012) |  |  |  |  |  | 36.89±19.36 |  | 166 |  |  | 55% |  |
| Newman^[14]^ | Australian | rs1801968, rs2296793, rs1182, rs3842225, rs13283584, rs11787741, rs2287367, rs1043186, rs13297609 | No | No | sporadic primary (focal, segmented, multifocal and generalised dystonia) | 63±14/ | 230 | 82/ | 68±11 | 228 | 84/ | Negative |
| (2012) |  |  |  |  |  | 46±15 |  | 148 |  |  | 144 |  |
| Groen^[24]^ | Dutch | rs1801968, rs2296793, rs1182, rs3842225 | No | Yes | primary cervical dystonia | 59.0±12.6/ | 367 | NA/ | 52.7±11.7 | 361 | NA/ | Negative |
| (2013) |  |  |  |  |  | 41.6±13.8 |  | 68.1% |  |  | 63.6% |  |
| Cheng^[41]^ | Chinese | rs1801968, rs2296793, rs1182, rs13297609 | Yes | Yes | early-onset primary dystonia (focal, segmental, multifocal and generalized) | 26.6±8.2/ | 121 | 59/62 | NA | 200 | NA/ | A statistically significant difference at frequencies of single alleles (OR=1.732, 95%CI =1.010–2.973, p=0.044) and genotypes (OR=1.623, 95%CI =1.100–2.395, p=0.015) of rs1801968 between patients with early-onset primary dystonia and normal subjects was detected. |
| (2013) |  |  |  |  |  | 21.1±7.4 |  |  |  |  | NA |  |
| Caputo^[33]^ | Argentinian | rs1801968 | No | Yes | primary focal, segmental, hemidystonic and generalized dystonia | NA/ | 40 | 19/ | NA | 200 | NA/ | Increased H216 frequency in all cases (p=0.0124) and in patients with positive family history (p=0.025) compared to controls |
| (2013) |  |  |  |  |  | 27.8±18.5 |  | 21 |  |  | NA |  |
| Timerbaeva^[40]^ | Russian (slavic and mixed) | rs1182, rs3842225 | No | Yes | primary focal, segmental including facial, cervical, laryngeal and arm muscles | NA/ | 254 | 93/ | NA | 225 | 92/ | Association at genotypic and allelic mode, for rs1182 (p=0.027, p=0.003) and for rs3842225 (p=0.017, p=0.002) with age of onset in Slavs. Only in Slavs statistical tendency for association of rs1182 with whole dystonia group (p=0.040) and with focal dystonia group (p=0.037) at genotypic model |
| (2015) |  |  |  |  |  | NA |  | 161 |  |  | 133 |  |
| Zhou^[20]^ | Chinese | rs2296793, rs3842225 | No | Yes | primary cervical dystonia | 41.25±15.76/ | 201 | 77/ | 43.35±14.77 | 289 | NA/ | Negative |
| (2015) |  |  |  |  |  | 38.27±16.06 |  | 124 |  |  | 59.9% |  |
| Wang^[23]^ | Chinese | rs1801968, rs2296793 | No | No | sporadic adult-onset primary focal dystonia (blepharospam, cervical dystonia, oromandibular dystonia, focal hand dystonia) | 54.00±11.62/ | 117 | 41/ | 59.36±10.40 | 131 | 67/ | Negative |
| (2016) |  |  |  |  |  | 53.23±11.77 |  | 76 |  |  | 64 |  |

SNP, single nucleotide polymorphism; CI, confidence interval; OR, odds ratio; NA, non-available.
