# Supplementary material for: The Role of TOR1A Polymorphisms in Dystonia: A Systematic Review and Meta-Analysis
Source: PLoS One. 2017 Jan 12;12(1):e0169934. doi: 10.1371/journal.pone.0169934 (PMC5231385; doi:10.1371/journal.pone.0169934)
Supplement: S2 Table — (DOCX) [file pone.0169934.s005.docx]

***S2 Table****: Characteristics of TOR1A SNPs that examined in the current meta-analysis.*

| **SNP** | **rs number** | **Chromosome position** | **Gene position** | **Function** | **MAF** |
| --- | --- | --- | --- | --- | --- |
| 1. | rs1801968 | 129818622 | exon 4 | Missence variant | 0.08 |
| 2. | rs2296793 | 129822779 | exon 2 | Coding synonymus | 0.23 |
| 3. | rs1182 | 129813781 | exon 5 | 3-‘UTR | 0.16 |
| 4. | rs3842225 | 129813148 | exon 5 | 3-‘UTR | 0.16 |
| 5. | rs13283584 | 129812583 | 3' downstream sequence | Downstream gene variant | 0.18 |
| 6. | rs11787741 | 129816005 | Intron 4-5 | Non-coding | 0.16 |
| 7. | rs13297609 | 129818407 | Intron 4-5 | Non coding transcript exon variant | 0.15 |

SNP, single nucleotide polymorphism; TOR1A, torsin1a; MAF, minor allele frequency; (http://www.ensembl.org/).
